# Beyond 30 Days: A Risk Calculator for Longer Term Outcomes of Prosthetic Breast Reconstruction

**DOI:** 10.1097/GOX.0000000000002065

**Published:** 2018-12-12

**Authors:** Jordan T. Blough, Michael M. Vu, Cecil S. Qiu, Alexei S. Mlodinow, Nima Khavanin, Neil A. Fine, John Y. S. Kim

**Affiliations:** From the * Department of Surgery, Division of Plastic and Reconstructive Surgery, Northwestern University Feinberg School of Medicine, Chicago, Ill.; † Department of Plastic and Reconstructive Surgery, Johns Hopkins Hospital, Baltimore, Md.

## Abstract

**Background:**

Despite growing use of surgical risk calculators, many are limited to 30-day outcomes due to the constraints of their underlying datasets. Because complications of breast reconstruction can occur well beyond 30 days after surgery, we endeavored to expand the Breast Reconstruction Risk Assessment (BRA) Score to prediction of 1-year complications after primary prosthetic breast reconstruction.

**Methods:**

We examined our prospective intrainstitutional database of prosthetic breast reconstructions from 2004 to 2015. Patients without 1-year follow-up were excluded. Pertinent patient variables include those enumerated in past iterations of the BRA Score. Outcomes of interest include seroma, surgical site infection (SSI), implant exposure, and explantation occurring within 1 year of tissue expander placement. Risk calculators were developed for each outcome using multivariate logistic regression models and made available online at www.BRAScore.org. Internal validity was assessed using C-statistic, Hosmer-Lemeshow test, and Brier score.

**Results:**

Nine-hundred three patients met inclusion criteria. Within 1-year, 3.0% of patients experienced seroma, 6.9% infection, 7.1% implant exposure, and 13.2% explantation. Thirty-day, 90-day, and 180-day windows captured 17.6%, 39.5%, and 59.7% of explantations, respectively. One-year risk calculators were developed for each complication of interest, and all demonstrated good internal validity: C-statistics for the 5 models ranged from 0.674 to 0.739, Hosmer-Lemeshow tests were uniformly nonsignificant, and Brier scores ranged from 0.027 to 0.154.

**Conclusions:**

Clinically significant complications of prosthetic breast reconstruction usually occur beyond the 30-day window following tissue expander placement. To better reflect long-term patient experiences, the BRA Score was enhanced with individualized risk models that predicted 1-year complications after prosthetic reconstruction (BRA Score XL). All models performed as well as, if not better than, the original BRA Score models and other popular risk calculators such as the CHA_2_DS_2_VASc Score. The patient-friendly BRA Score XL risk calculator is available at www.brascore.org to facilitate operative decision-making and heighten the informed consent process for patients.

## Abstract 2

Supplemental Digital Content is available in the text.

## INTRODUCTION

Individualized risk estimation tools have become powerful aids on the medical wards, facilitating rapid decision-making at the bedside with the input of patient-specific data. Prominent examples include the CHADS2VASc score, which accounts for age, sex, and stroke history to predict the annual risk of stroke in patients with atrial fibrillation;^1^ and the Caprini assessment of venous thromboembolism risk, used to select appropriate postoperative venous thromboembolism prophylaxis in both general and plastic surgical patients.^2,3^ Surgical fields have also developed risk calculators that can take patient-specific inputs and generate absolute risk estimations for a variety of complications.^4–10^ Risk calculators serve not only to facilitate a surgeon’s patient selection and perioperative optimization, but also to empower patients by providing personalized and comprehensible absolute risks.

The universal American College of Surgeons National Surgical Quality Improvement Program (ACS-NSQIP) Risk Calculator was released in 2013 and was subsequently followed by several specialty- and procedural-specific calculators that outperformed the universal ACS-NSQIP calculator and have since been adopted in their respective fields.^5–16^ Few of these devices currently exist specifically for plastic surgeons. Our group introduced the first iteration of the Breast Reconstruction Assessment (BRA) Score in 2014 to predict risk of 30-day complications for women undergoing immediate autologous or prosthetic breast reconstruction.^17–19^ Available to surgeons and patients through an open-source, patient-centric, online platform at www.brascore.org, its flexible underlying design permits additions and adjustments to the core risk calculator as new data become available for analysis. BRA Score has since been externally validated against a large intrainstitutional cohort for 30-day complications occurring after prosthetic reconstruction.^20^

Thus far, both the universal ACS-NSQIP risk calculator and BRA Score have been limited to predicting complications only out to 30 days. This time horizon is emphasized in publicly reported quality metrics, explaining why many risk calculators are limited to it. The 30-day window is sensible for some surgical procedures; however, complications of breast reconstruction—such as surgical-site infection (SSI), flap necrosis, seroma, and reconstructive failure—are known to occur well beyond 30 days.^21–28^ Therefore, while institutions are typically only required to report on their 30-day complications, we must go further for our breast reconstruction patients to accurately prognosticate and discuss their operative risk, and improve surgical decision-making. The aim of this study was to extend the BRA Score risk calculations for complications of immediate prosthetic breast reconstruction occurring up to 1 year postoperatively. Using a large, intrainstitutional cohort of patients with long-term follow-up, we developed the BRA Score: Extended Length (XL).

## METHODS

### Data Collection

This study was approved by the Northwestern University Institutional Review Board. A query of our prospectively collected intrainstitutional database was performed for all, consecutive, immediate 2-stage reconstructions occurring between 2004 and 2015. Breast reconstructions were performed by the senior authors (J.Y.S.K. and N.A.F.). Inpatient and outpatient records were reviewed for each patient to obtain the pertinent demographics, perioperative characteristics, postoperative complications, and follow-up. Patients were excluded if lost to follow-up before 1 year.

### Perioperative Variable Selection and Outcomes

We examined the same perioperative variables of interest that were incorporated in the original iteration of BRA Score. These variables had been selected based on their likely association with complications, using the existing literature and our own clinical experience.^23–26,29–36^ This “interactive model-building approach” to risk modeling is superior to statistically automated selection, which is typically less stable and less reproducible.^37^ Variables included age, body mass index (BMI), American Society of Anesthesiologists’ (ASA) class, whether the patient smoked within a year before surgery, pulmonary comorbidities, hypertension requiring medication, peripheral vascular disease/coronary artery disease, diabetes (both insulin-dependent and non–insulin-dependent), prior percutaneous coronary intervention/cardiac surgery, bleeding disorder or receiving chronic anticoagulation, bilateral versus unilateral reconstruction, pre- or postoperative radiation therapy, and chemotherapy therapy.

Primary outcomes of interest were complications that are accounted for in previous iterations of the BRA Score.^17–19^ Outcomes included 1-year occurrences of clinically assessed seroma, SSI (superficial, deep, and organ-space), dehiscence/expander exposure (defined as fascial separation of the surgical wound), and unplanned expander explantation following stage I tissue expander placement. Planned expander removals and exchanges were not considered complications. These were combined to create a pooled 1-year surgical complication variable. Outcomes were tracked by their postoperative timing and recorded based on occurrence within 1-, 3-, 6-, and 12-month postoperative windows.

### Statistical Analysis

Missing values were extrapolated with multiple imputation using fully conditional specification under the assumption of values missing at random. Imputation modeling used all aforementioned perioperative and outcome variables. Twenty imputations were performed and pooled, as this number has been found to optimize efficiency and reproducibility of results.^38^

Associations between the perioperative variables of interest and the 1-year complications were tested with univariate analysis; Pearson’s chi-square or Fischer’s exact tests were used for categorical variables and Mann-Whitney *U* test was used for continuous variables.

Five multiple logistic regression models, 1 for each 1-year outcome of interest and 1 for any complication at all, were generated according to the aforementioned perioperative covariates to compute probabilities of experiencing an outcome. When a covariate was unable to be reliably incorporated in a model, we excluded it from that particular model.

Each model was internally validated via C-statistic, Hosmer-Lemeshow (H-L) test, and Brier score, as performed previously in the initial iteration of the BRA Score.^15,17,19^ The C-statistic is the area under the receiver-operating curve and measures model discrimination, for which 0.5 is equivalent to a coin-flip and 1.0 is perfect discrimination. The H-L test measures calibration (goodness-of-fit). Brier scores indicate overall model accuracy based on the difference between predicted and observed outcomes, with 0.0 representing no difference and thus perfect accuracy. Each metric has its strengths and limitations in assessing model performance. Taken together, these 3 validation metrics offer the most comprehensive measurement, each filling in the gaps of the others.^5,39–41^ Statistical analyses were performed using SPSS version 23 (IBM, Armonk, N.Y.).

### Modification of the Online Risk Calculator Platform

Each risk model was added to the preexisting BRA Score interface, extending output of complications to 1-year postoperatively for prosthetic-based breast reconstruction. The newly upgraded BRA Score XL is available open-access to patients and surgeons alike at BRAScore.org. Its user-friendly design enables users to input known perioperative information into the fields, returning probability estimates for each complication that are tailored to the patient.

## RESULTS

### Cohort Characteristics and Outcomes

A total of 1,266 women who underwent TE/implant-based reconstruction were reviewed in our database, 903 of which met final inclusion criteria. Of these women, 49.6% had bilateral reconstruction and 50.4% had unilateral reconstruction, representing a total of 1,365 breasts. Median follow-up length was 22.3 months. Patient demographics are illustrated in Table 1. Mean age was 49.3 years (SD, 10.6) and mean BMI 26.6 kg/m^2^ (SD, 6.0). Importantly, 41% of women received radiation therapy—9.3% prereconstruction versus 31.7% postreconstruction. Over half (53.0%) underwent chemotherapy infusion. Notably, 10.3% of patients had ASA classes of 3 or greater, 24.1% were smokers within the previous year, 18.6% had hypertension requiring medication, 10.5% had a bleeding disorder or were receiving chronic anticoagulation, and 4.5% had diabetes. Cardiopulmonary comorbidities carried by the cohort included peripheral vascular disease or coronary artery disease (peripheral vascular disease/coronary artery disease, 5.0%), dyspnea (4.5%), and history of percutaneous cardiac intervention or cardiac surgery (1.3%).

**Table 1.**
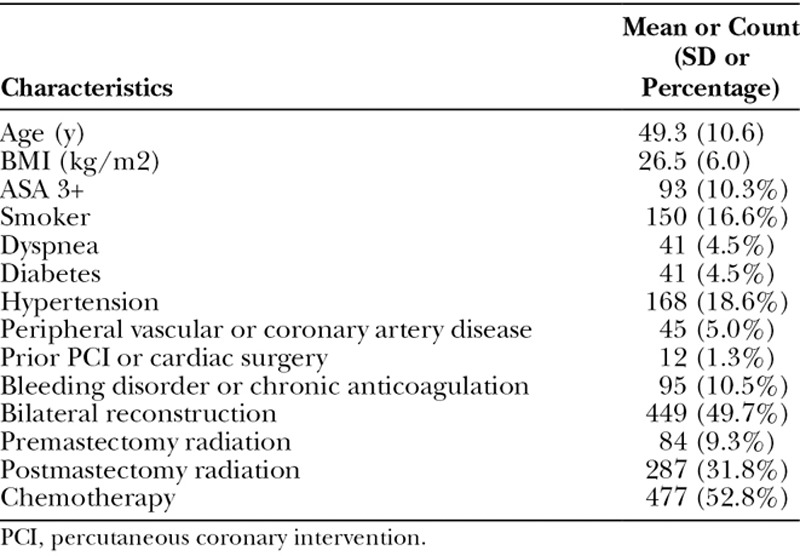
Patient Demographics and Clinical Traits

**Fig. 1.**
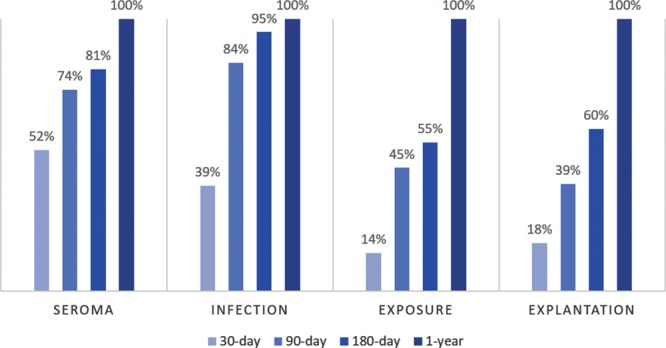
Cumulative percentage of events observed with extended observation periods. The 30-day observation period captured a minority of all events, capturing 52% of seromas, 39% of infections, 14% of exposures, and 18% of explantations. Exposures and explantations tended to occur especially late, with 45% and 40% occurring beyond 180 days, respectively.

The 1-year complication rates of the cohort are depicted in Table 2. The most common complication at 1-year of follow-up was explantation of the implant (13.2%), followed by implant exposure (7.1%), infection (6.9%), and seroma (3.0%). Each complication type varied in terms of when it presented on average, which can be appreciated by 30-day, 90-day, 180-day, and 1-year periods of observation in Figure 1. The 30-day observation period captured a minority of all events, capturing 52% of seromas, 39% of infections, 14% of exposures, and 18% of explantations. Exposures and explantations tended to occur especially late, with 45% and 40% occurring beyond 180 days, respectively.

**Table 2.**
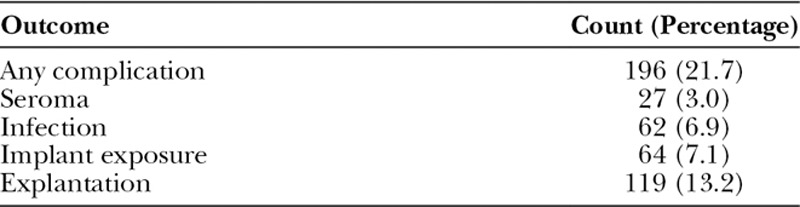
Complications Observed by 1-year Follow-up in Our Sample

### Risk Modeling and Model Performance

**Fig. 2.**
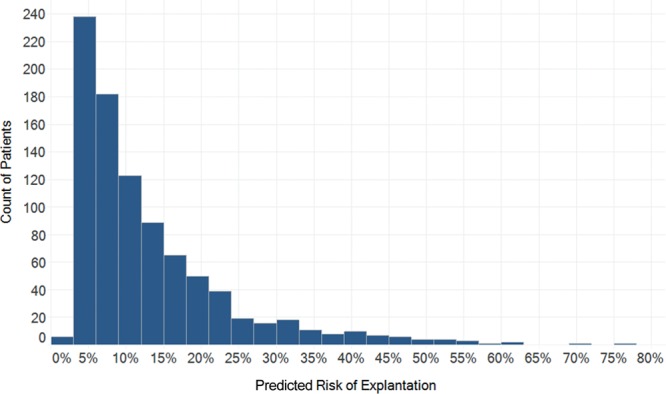
The distribution of predicted risk of explantation within 1 year, estimated by the BRA Score XL model. Mean risk was 13.2% while the median risk was 9.4%. The distribution was wide (SD = 10.8%) and significantly positively-skewed (skewness = 2.027), implying that most patients are at lower risk than a population average would suggest. Indeed, two-thirds (65%) had an estimated risk less than the mean.

Five risk models were developed in accordance with the selected perioperative variables, 1 for each complication of interest at 1-year follow-up (seroma, exposure, infection, and explantation) and a composite variable capturing the occurrence of any of those complication. All perioperative variables were included in each logistic regression model unless their beta coefficients yielded erratic results, in which case they were excluded from the regression. For example, having a high ASA classification and having hypertension were paradoxically found to be protective of several complications, so they were both excluded from all models. Smoking status and history of cardiac intervention were similarly excluded from the seroma model, and diabetes was excluded from the exposure model. The beta coefficients for each covariate of the final regression models are presented in Table 3. Figure 2 includes the distributions of predicted risk for explantation as an example, illustrating a wide distribution of risk around the mean. The distribution of risk is also notably positively skewed, indicating that high-risk outliers have shifted the mean risk to be greater than the median risk for the population.

**Table 3.**
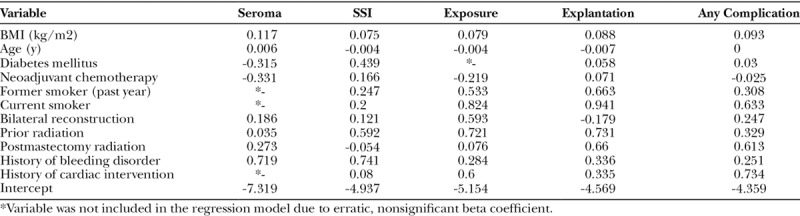
Beta Coefficients of Each BRA Score XL Regression Model

**Fig. 3.**
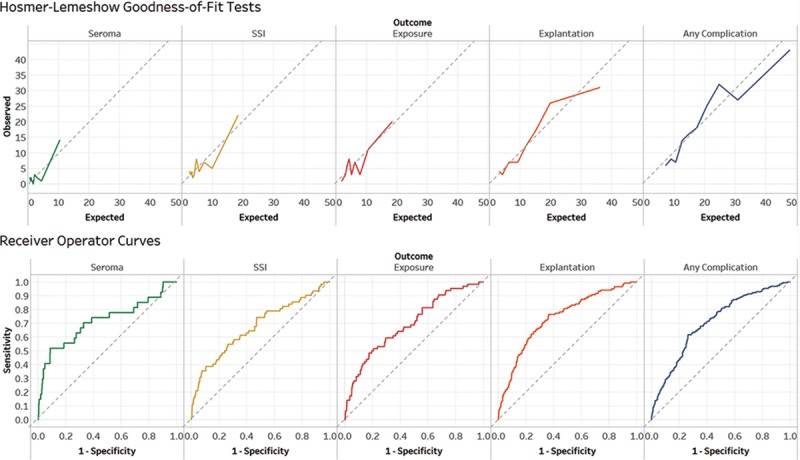
Graphical representation of H-L goodness-of-fit test and C-statistics (equivalent to the area under the receiver-operator curve). Charting expected vs observed rates of complications demonstrated good agreement between number of predicted and observed events. Indeed, the H-L tests for each model were nonsignificant (ie, greater than 0.05), indicating minimal deviation from the diagonal reference line of perfect agreement (dotted gray). C-statistics ranged from 0.674 for infection to 0.739 for explantation, indicating good discrimination or ability to distinguish high- and low- risk patients.

All 5 models yielded acceptable calibration, discrimination, and accuracy based on the C-statistic, H-L test, and Brier score (Fig. 3, Table 4). C-statistics ranged from 0.674 for infection to 0.739 for explantation, indicating good discrimination or ability to distinguish high- and low- risk patients. The H-L tests for each model were nonsignificant (ie, greater than 0.05), indicative of good calibration, meaning that there was good agreement between number of predicted and observed events. Finally, Brier scores were low (range, 0.027–0.154), indicating good model accuracy.

**Table 4.**
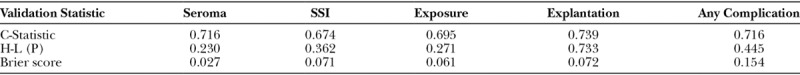
Key Statistics for Internal Validation of BRA Score XL Models

### Online Risk Calculator

**Fig. 4.**
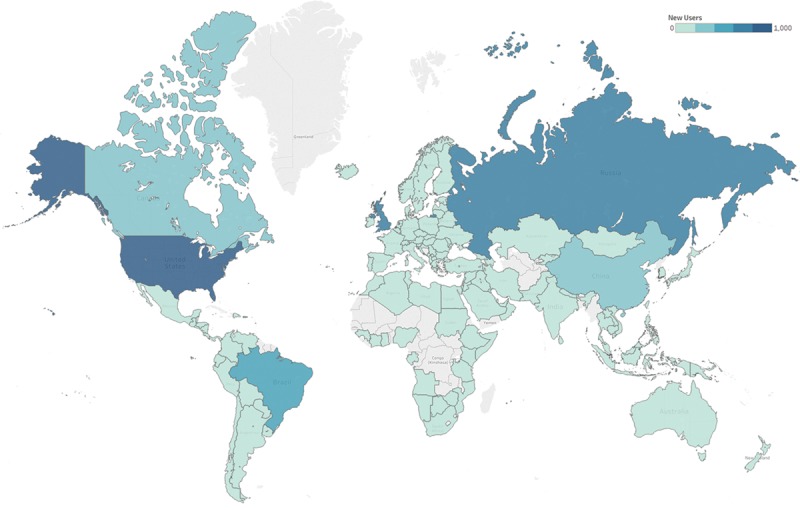
Heatmap demonstrating number of unique viewers of the BRA Score website (www.brascore.org) per country since 2014 launch. Darker blues indicate more users on absolute scale.

**Fig. 5.**
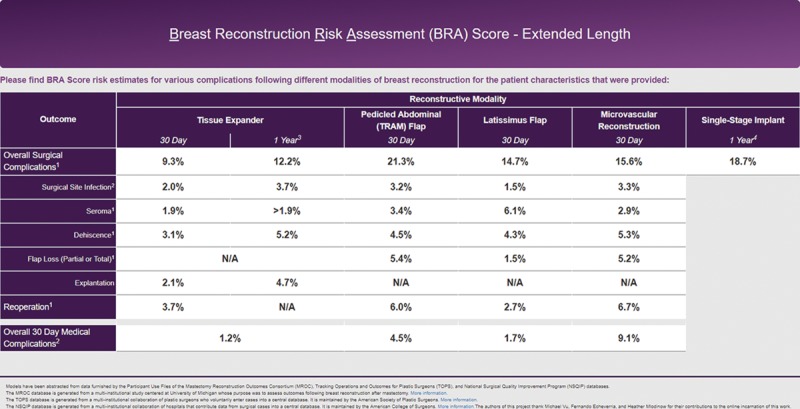
Example web browser output from www.BRAScore.org for a 40-year-old woman with a BMI of 23 kg/m^2^ and no comorbidities undergoing bilateral reconstruction.

**Fig. 6.**
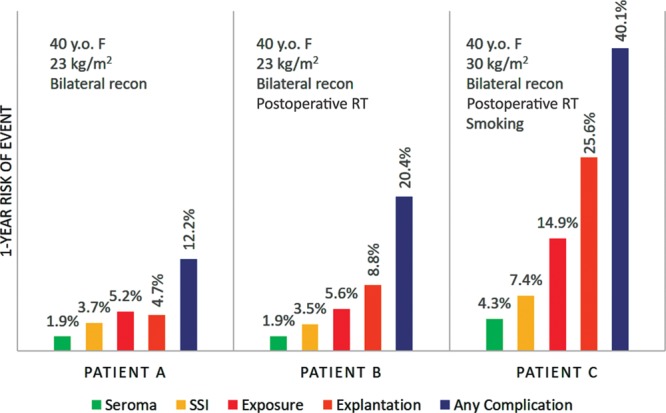
Example output of the BRA Score XL for 3 patients with sequentially worse risk profiles. Patient A: 40-year-old woman with a BMI of 23 kg/m^2^ and no comorbidities undergoing bilateral reconstruction. Patient B: same as patient A, except that she receives postoperative radiation therapy. The risk of explantation nearly doubles. Patient C: same as patient B, except now she has a BMI of 30 kg/m^2^, has smoked within the past year, and still receives postoperative radiation therapy. The risk of explantation within a year is 26%.

Since its introduction in 2014, the online BRA Score (www.brascore.org) risk calculator has been used by over 9,800 visitors, approximately one-third of which are international users from all over the world (Fig. 4). Average session duration is 3:30 minutes per visitor, indicating meaningful use. As of June 2018, our existing iteration of the BRA Score has been newly modified to incorporate 1-year risk of explantation, exposure, infection, seroma, and overall risk of complication for expander-based reconstruction. The user interface has been updated, and an example of its usage may be seen in **Supplemental Digital Content 1** and example output displayed in Figure 5 (see video, Supplemental Digital Content 1, which displays usage of the risk calculators at www.BRAScore.org. This video is available in the “Related Videos” section of the Full-Text article at PRSGlobalOpen.com or at http://links.lww.com/PRSGO/A933). Risk estimates displayed graphically in Figure 6 illustrate how the BRA Score XL models incorporate patient criteria to predict operative risk for 3 hypothetical patients A, B, and C of sequentially worsening risk profiles. Patient A is a 40-year-old woman with a BMI of 23 kg/m^2^ and no comorbidities. Patient B has the same traits as patient A, except that her treatment plan includes postoperative radiation therapy. The risk of explantation nearly doubles from 4.8% to 8.8%. Patient C is a 40-year-old woman who has a BMI of 30 kg/m^2^, has smoked within the past year, and plans to undergo postoperative radiation therapy. The risk of explantation within a year is 26%, and the risk of any complication at all exceeds 40%.

**Video Graphic 1.**
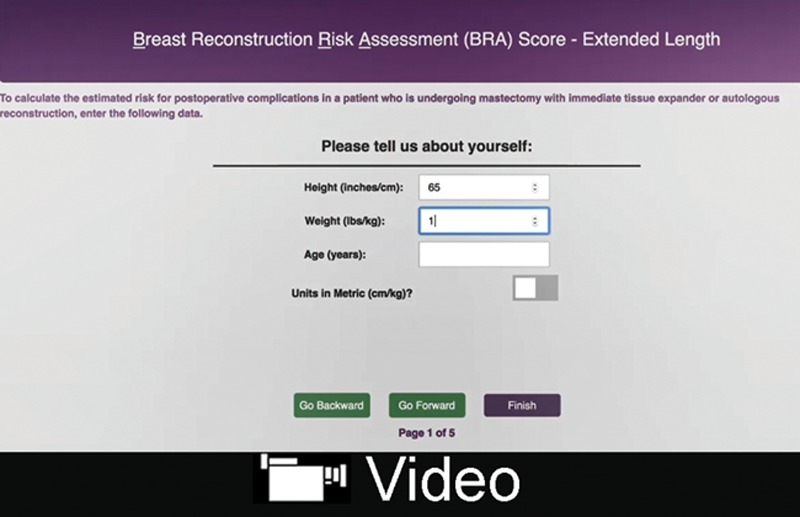
See video, Supplemental Digital Content 1, which displays usage of the risk calculators at www.BRAScore.org. This video is available in the “Related Videos” section of the Full-Text article at PRSGlobalOpen.com or at http://links.lww.com/PRSGO/A933.

## DISCUSSION

After the Women’s Health and Cancer Rights Act of 1998 guaranteed that reconstruction and subsequent revisionary procedures are covered by healthcare payers for women diagnosed with breast cancer, breast reconstruction procedures saw over an 80% increase in the following decade alone.^42,43^ Approximately 40% of women who undergo mastectomy choose to undergo immediate reconstruction, the large majority of which are TE/implant-based techniques.^43^ American Society of Plastic Surgeons members performed over 109,000 alone in 2016.^44^ Although for many women breast reconstruction is in fact a requirement for psychosocial well-being, it is not medically necessary.^45^ Studies of patient surveys report that major concerns regarding breast reconstruction include fear of complications, reconstructive failure, and necessity of subsequent revisions or reoperations.^46–48^ Furthermore, when surveyed about their perceived risk of experiencing any complication whatsoever, a plurality of patients underestimate mean risk.^49^ Thus, judicious patient selection, surgical approach, and preoperative counseling that sets clear expectations are paramount to optimize outcomes perspective and patient satisfaction. Risk calculators can serve as a valuable counseling tool for surgeons to help patients avoid misunderstandings and better grasp their individual risk. To that end, the first iteration of the BRA Score was released in 2014 to offer a solution to this problem.^17–19^ Since then, the BRA Score has provided valuable information to over 10,000 users across the globe (Fig. 4).

At its conception, the BRA Score was envisioned to be flexible and modifiable so that it could be updated as new data become available. In addition to its original internal validation for 30-day complications, it has recently been externally validated against a separate cohort.^20^ In this study, we further expanded the clinical utility of the original BRA Score by developing new risk equations for prosthetic breast reconstruction that calculate individualized chance of 1-year complications, collectively called the BRA Score XL. BRA Score XL captures a greater swath of possible complications in a more clinically relevant timeframe.

This cohort demonstrated the inadequacy of the 30-day timeframe for all our complications of interest, especially explantation. In our investigation, 82% of explantations manifested beyond 30 days (Fig. 1) with median time to occurrence of 192 days. Further, 30-day, 90-day, and 180-day windows only captured 17.6%, 39.5%, and 59.7% of explantations, respectively (Table 3). Of all complications, explantation is particularly unlikely to occur within the first 30 days because surgeons typically opt to observe and manage whatever complication may be threatening explantation and attempt to salvage the reconstruction. In addition to explantation, exposure and SSI tended to surface beyond 30 days in our study. Only 39% of SSIs and 14% of exposures were captured within 30 days. Finally, almost half (48%) of patients who developed seromas presented beyond 30 days, with 19% still arising within the 180- to 365-day window. Taken together, these findings reinforce our obligation to extend the BRA Score to a complete year of follow-up, as the 30-day period would appear to greatly underestimate risk.

Other authors have reported similar timescales along which these complications occur. In an analysis of 1,662 implant-based reconstructions in 1,024 patients out of the Mastectomy Reconstruction Outcomes Consortium Study, 47–71% of all SSIs occurred beyond the 30-day window. Nearly 44% of these late SSIs required explantation in comparison to 26% of early SSIs (within 30 days), showing the greater likelihood of explantation during later timeframes.^23^ A retrospective study of secondary implant reconstruction by Spear et al.^21^ reported that explantations occurred a mean of 262 days after expander placement, and that 66% were indicated because of infection. A similar study by Halvorson^22^ noted an average removal time of 56 days postoperatively (range, 7–211 days).

One-year risk of complications in our cohort are comparable to other long-term follow-up values reported in the literature (Table 2).^23–26,50–52^ Although these rates are useful as benchmark figures for the population, they have limited utility as risk estimates for most individual patients. That is, the mean incidence of a complication is not representative of most patients, because the distribution of risk is so positively skewed (skewness = 2.027, Fig. 2). As the high-risk outliers stretch the mean to be greater than the median, it becomes apparent that for most patients (median) the population-based estimate (mean) largely overestimates their risk. Indeed, two-thirds of patients had an estimated risk of explantation less than the mean (13.2%). As a correlate, it becomes crucial to identify these high-risk outliers, which can be attempted using clinical judgment based on known risk factors in the literature including smoking, higher BMI, hypertension, radiotherapy, immediate reconstruction, and older age.^23–26^ However, objectively quantifying the magnitude of impact of even one risk factor is troublesome to achieve. Furthermore, for each unique patient with her own unique risk profile blueprint, it becomes even more arduous to quickly and accurately predict the magnitude and interplay of multiple simultaneous risk and/or protective factors.

To demonstrate the utility of risk calculators, we have illustrated how risk varies between hypothetical patients with different profiles (Fig. 5). The average 1-year risk for explantation in our cohort was 13.2%, yet for an otherwise healthy, normal-BMI, 40-year-old woman, her absolute individualized risk is in fact under 5%—a failure rate much less likely to deter her from pursuing reconstruction. In the next example, in which this same woman must now undergo radiotherapy, a known factor predisposing her to failure, her risk of explantation almost doubles to 8.8%, a risk multiplier consistent with past reports.^52,53^ When further comorbidities are added, the benefits of precision risk calculation become further evident. A BMI of 30 kg/m^2^ and smoking status are added in addition to radiotherapy, causing her 1-year risk of explantation to rise to 26% and risk of any complication at all exceeds 40%, which may be sufficient reason for her to consider alternatives such as autologous reconstruction.

Each model underlying the risk calculator demonstrated sufficient internal validity as measured by the C-statistic, H-L test, and Brier score (Fig. 3, Table 4). C-statistics of each model (range, 0.674–0.739) demonstrated good discriminatory capacity, comparable to the widely-used CHADS2VASC score (C-statistic = 0.647) and the first BRA Score iteration (C-statistic range 0.623 - 0.685).^1,17–19^ Goodness-of-fit for each model was ascertained with nonsignificant H-L tests. Finally, the models’ Brier scores, which describe calibration in addition to discrimination, also fared well compared with existing tools including the ACS NSQIP Universal Risk Calculator and the original BRA score (Brier Score range, 0.032–0.128).^5,17–19^

Our study fills an important gap in the prior iteration of the BRA Score and has the strength of a large sample of patients shared between 2 different surgeons at our institution. There do remain important limitations to address. Unlike the original 30-day BRA Score, which was developed using multi-institutional data, the BRA Score XL is based upon a large albeit single-institution experience. Therefore, it may be less generalizable to other surgeons who see different patient populations, use different techniques, operate in facilities with different protocols and resources, and possess different experience levels. Our models, which include an ambitious number of patient factors, also are at risk of overfitting to our limited sample, which we mitigated by eliminating variables that behaved erratically. Complication rates in this study are indeed within literature ranges, but future work may involve modifying risk models to include cases performed by additional surgeons. Future efforts may also include adding an option for surgeons to adjust the risk models according to their own personal observed, average complication rates. Additionally, BRA Score does not account for poor long-term aesthetic outcomes such as rippling, malposition, capsular contracture, and unsatisfactory scarring—each responsible for complicating a substantial portion of implant-based reconstructions.^54^ In particular, it will be essential to predict the requirement for revisionary procedures secondary to these adverse aesthetic outcomes to truly inform patients of what the reconstructive process may entail. Furthermore, patient-reported outcomes would be an important extension in predicting satisfaction with surgery, achievable through validated instruments such as the BREAST-Q.^55,56^ Our immediate future directions for BRA Score are to perform a similar extension of the timeframe of risk calculation for autologous reconstruction as well, where late complications such as fat necrosis and incisional hernia will be important outcomes to predict.

## CONCLUSIONS

The BRA Score was released in 2014 to be a modifiable risk assessment tool for 30-day complications in breast reconstruction, compiling unique patient factors to provide accurate and quantitative risk assessment. As a continuation of this work, the authors developed and validated a calculator extending risk assessment to a full year, thereby capturing previously unaccounted-for long-term risk such as late SSI, exposure, and explantation. The patient-friendly BRA Score XL risk calculator is available open-source at www.brascore.org to facilitate operative decision-making and heighten the informed consent process for patients.

## Supplementary Material
